# *In vitro* Evaluation of Cytotoxicity, Biocompatibility, and Osteogenic Effect of Two Calcium Silicate Sealers Compared with AH Plus Jet

**DOI:** 10.1055/s-0044-1789602

**Published:** 2025-03-12

**Authors:** Monique Aparecida de Lima Rios Pitzschk, Carlos Eduardo da Silveira Bueno, Carlos Eduardo Fontana, Alexandre Sigrist De Martin, Daniel Guimarães Pedro Rocha, Carolina Pessoa Stringheta, Ana Grasiela da Silva Limoeiro, Virgilio Marcio Bastos Braga, Caroliny Chavier Guimaraes, Marilia Fagury Videira Marceliano-Alves, Wayne Martins Nascimento, Thiago Sena Guimarães, Rina Andrea Pelegrine

**Affiliations:** 1 Faculdade São Leopoldo Mandic, Instituto de Pesquisa São Leopoldo Mandic, Campinas, São Paulo, Brazil; 2 PUC-Campinas, Center of Life Sciences, Postgraduate Program in Health Sciences, Campinas, São Paulo, Brazil; 3 Pontifical Catholic University of Campinas, Department of Endodontics, School of Life Sciences, Campinas, São Paulo, Brazil; 4 Department of Restorative Dentistry, Dental Materials, and Endodontics, Bauru School of Dentistry, University of São, Bauru, Brazil; 5 Postgraduate Program in Dentistry, Iguaçu University, Nova Iguaçu, RJ, Brazil; 6 Mauricio de Nassau University Center, Dentistry Department, Rio de Janeiro, Brazil; 7 Department of Dental Research Cell, Dr. D. Y. Patil Dental College and Hospital, Dr. D. Y. Patil Vidyapeeth, Pune, India

**Keywords:** calcium silicate, epoxy resins, osteogenesis, root canal filling materials

## Abstract

**Objective** The purpose of the study was to investigate the cytotoxicity, biocompatibility, and osteogenic effect of EndoSequence BCSealer HiFlow (BCH) and Bio-C Sealer (BCS) in osteosarcoma cells SAOS-2 compared with AH Plus Jet.

**Materials and Methods** For cytotoxicity analysis, the [3-(4,5-dimethyl-thiazole)-2,5-diphenyltetrazolium bromide-MTT; Sigma/Aldrich] method was used after 24, 48, and 72 hours. For cellular bioactivity, alkaline phosphatase enzyme (ALP) was evaluated after 7 and 14 days.

**Statistical Analysis** MTT assay was assessed using analysis of variance (ANOVA) and Tukey’s test and over time using ANOVA and Sidak’s test. For ALP analysis, the one-way ANOVA and Student’s paired t-tests were used.

**Results** BCS was like AH Plus Jet. After 72 hours, BCH and BCS did not differ from AH Plus Jet and showed similar behavior in terms of viability. ALP showed a difference only after 14 days. BCH had the lowest bioactivity value. AH Plus Jet and BCS were like each other.

**Conclusion** AHP was more viable for SAOS-2, and the biocompatibility of Calcium silicate-based sealers was acceptable. The present finding assessed the biocompatibility of Calcium silicate bioceramic sealers, such as BCS, EndoSequence BCH, and AH Plus Jet, is acceptable and they have osteogenic effect and bioactivity.

## Introduction

For the root canal system (RCS) obturation, the main technique is the gutta-percha lateral or vertical compaction. 1 These must have as desirable properties the ability to seal the root canal in lateral and apical aspects, adhere to the dentin walls, be easy to insert, not stain the tooth structures, have a good working time, be bactericidal or bacteriostatic, and, when extruded, be well tolerated by periapical tissues. 1 One goal in root canal obturation procedure is to keep the filling material within the RCS to avoid exacerbating inflammatory processes in the periapical tissues. 2

Calcium silicate bioceramic sealers (CSBS) have been introduced in clinical practice mainly because of their biocompatibility, antibacterial activity, and osteogenic potential. Biocompatibility is a desirable property for endodontic sealers and is directly related to the composition of the material. 3 For ready-to-use premixed obturation sealers such as Bio-C Sealer (BCS, Angelus, Londrina, Brazil) and EndoSequence BCSealer HiFlow (BCH, Brasseler, Savannah, United States), the residual moisture in the root canal together with the dentin moisture should provide the water required for hydration of the material. 4

The EndoSequence BCH (Brasseler) has garnered attention among new root canal sealers. A previous study assessed its clinical outcomes in nonsurgical root canal treatments, revealing an impressive success rate of 90.9%. 5 This sealer presents notable properties, such as antibacterial effect, biocompatibility, adequate sealing, promotes the dentin adhesion, and undergoes slight expansion, unlike traditional sealers such as AH Plus Jet and Pulp Canal Sealer, which experience shrinkage. 6 This sealer also has radiopacity, hydrophilicity, and antibacterial properties. Its composition, based on calcium phosphate silicate, leads to the formation of hydroxyapatite when exposed to water, further enhancing the bond between the sealer and dentin. 7 8

The BCS, a bioceramic root canal sealer developed by Angelus Science and Technology, is another silicate bioceramic sealer contain components with antimicrobial properties, because when in contact with bacteria, the calcium silicate contained in the sealer releases calcium hydroxide ions that create an alkaline environment, which disrupts the bacterial cellular processes, inhibiting their growth and promoting antimicrobial effects. 9 10 11 This sealer also has low solubility (above 3%) and its volumetric loss is above 0.1%. 12 It combines a mixture of calcium and magnesium silicate, calcium sulfate, potassium sulfate, zirconium oxide, and silica. This unique composition enables a prolonged release of calcium ions and enhances its biocompatibility with living tissues. 13

The AH Plus Jet (Dentsply De Trey, Konstanz, Germany) is resin-based sealer that uses an automatic double-barrel syringe that precisely mixes the paste-to-paste formula in a 1:1 ratio, eliminating the need for manual mixing. Based on epoxy–amine resin, it has long-term sealing and self-adhesive properties, broad dimensional stability, and radiopacity. 14

Laboratory studies are being conducted to evaluate the biocompatibility and viability of materials in different areas of endodontic therapy. To date, one of the most used methods is the [3-(4,5-dimethyl-thiazole)-2,5-diphenyltetrazolium bromide—MTT; Sigma/Aldrich] assay, which can replace laboratory animal models in many cases due to its versatility, simplicity, accuracy, and time savings. 15

A comparative analysis allows for direct comparisons of the antimicrobial properties of various bioceramic sealer. This valuable information aids clinicians in choosing the most appropriate bioceramic sealer for specific clinical situations depending on the spectrum of antimicrobial activity and compatibility with other endodontic materials and techniques. Therefore, the aim of this work was to investigate *in vitro* the cytotoxicity and osteogenic potential of BCH and BCS compared with AH Plus Jet in osteoblastic cells. The null hypothesis tested was that the sealers would be similar with respect to the parameters evaluated.

## Materials and Methods

A power calculation will be performed using the G*Power 3.1 software (Heinrich Heine University, Dusseldorf, Germany) using a power β = 95% and α = 5% and an independent sample test will be applied. The test samples consisted of cement disks measuring 5mm × 2mm in diameter. After the setting time, it was observed that the samples were not uniform; therefore, following the ISO 10993 standard, as mentioned, the first step for obtaining the extracts was weighing to determine the appropriate volume of extract for each cement. The experiment itself was conducted in triplicate, meaning that three independent experiments were performed in triplicate for each experimental group.

This study protocol was accepted by the local Ethics and Human Research Committee (CAAE number: 3746621.8.0000.5374). SAOS-2 osteoblast cells were seeded and maintained in a CO _2_ oven to provide an appropriate environment. The culture medium was renewed every 2 days to ensure nutrient supply and allow proliferation. Cell growth was monitored with an inverted phase microscope, and morphology, adherence, and proliferation were also monitored. Plating was performed in 96-well plates and with complete osteogenic medium (McCoy 5A with L-glutamine supplemented with 10% fetal bovine serum, 7 mmol/L β-glycerophosphate, 5 µg/mL ascorbic acid, and 50 µg/mL gentamicin).

Before plating, the cells had to be counted, for which the Neubauer chamber was used. For this count, a glass slide was placed over the chamber containing the cell suspension. The spacing was 0.1 mm and the volume of each resulting square was equal to 0.1. To get the cells onto these glass coverslips, 100 µL of trypsin was first added to the well of the plate, and from this 100 µL, 1 mL of medium (10 µL of cells) was added to a Petri dish using a pipette. Eight drops of trypan blue were added, corresponding to 10 µL, and then the mixture was pipetted into each layer (slide) of the Neubauer chamber. The cell count was read in the inverted phase microscope and 110 cells/mm ^2^ per well (0.42 × 10 ^4^ ) were obtained for plating. Immediately after counting the SAOS cells, plating was performed in a 96-well cell culture plate with complete medium, as mentioned previously. These cells remained in the incubator at 37°C and 5% CO _2_ for 24 hours.

### Preparation of the Samples for the Extracts Obtention

To prepare the samples, an alginate model was made in order to produce an acetate tray using acetate sheets (Bio Art Equipamentos Odontológicos, São Carlos, Brazil) with a vacuum plasticizer, which served as a costumed tray, with 15 holes, for the sealers (AH Plus Jet, BCH, and BCS) placement using their own syringes. For CSBC that require moisture from the outside, a moist sterile gauze was left under the mold pressed with glass plates for the first 24 hours. After this time, the gauze was removed, and the sealers were left for another 24 hours. A total time of 48 hours were necessary to until the sealers completely set. 7 16 17

### Preparation of the Extracts

Using a precision balance (FS-120 iPesage, Cabestany, France), the sealers were weighed individually to determine the sample weight to set the ideal volume of medium for each extraction, as shown in Table 1 . Inside a laminar flow chamber, the Petri dishes containing the samples were irradiated with ultraviolet light for 15 minutes for sterilization. 7

**Table 1:** Volume of extract as a function of sample weight

Sample	Weight (g)	Volume of medium (mL)
AH Plus Jet	2.490	12.45
Bio-C	1.742	8.71
HiFlow	1.413	7.065

After irradiation of the samples with ultraviolet light, the sealers were transferred to 15-mL tubes containing the appropriate volume of medium for each sample. The extraction medium used was McCoy 5A culture medium (Sigma-Aldrich, Saint Louis, United States) containing L-glutamine supplemented with 10% fetal bovine serum, 7 mmol/L β-glycerophosphate, 5 µg/mL ascorbic acid, and 50 µg/mL gentamicin (osteogenic medium-complete). The tubes containing the samples were incubated at 37°C and 5% CO _2_ for 24 hours to obtain the extracts. The groups were divided into Control group: SAOS cells cultured in complete medium; APH group: cells cultured in conditioned medium with AH Plus Jet; BCS group: cells cultured in conditioned medium with BCS; BCH group: cells cultured in conditioned medium with EndoSequence HiFlow.

**Fig. 1:**
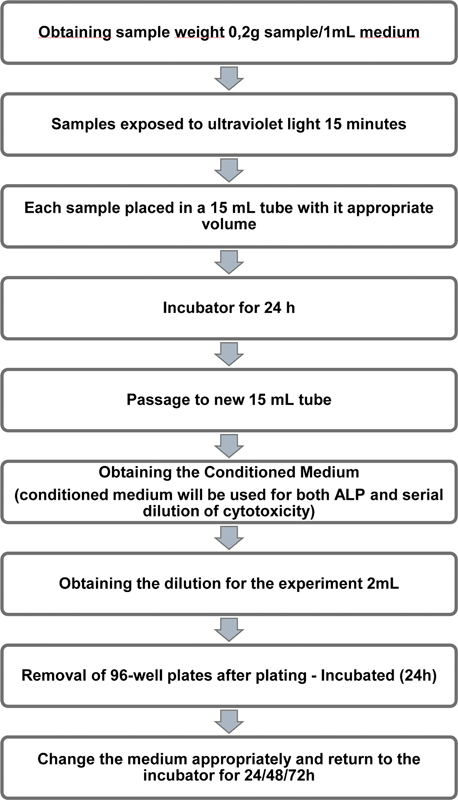
Preparation of eluates for the tests MTT and alkaline phosphatase (ALP) assays.

After 24 hours, the tubes containing the sealers were transferred to another tube containing 15 mL to obtain the extract. The tube of BCH was centrifuged at 20°C, at 5.000 rpm for 7 minutes, because it was turbid in the medium since the sealer had not reached the expected hardening. After the three tubes were filtered with pipettes or transferred to new 15-mL tubes, the dilution was counted for this experiment and a 1:5 dilution was made with four parts osteogenic medium, and one part to conditioned medium. The samples preparation flowchart for obtaining the extracts for the tests can be found in Fig. 1 .

After 24 hours, the cells were first observed in the 96-well plates of the incubator and in an inverted phase microscope. Then, the medium conditioned with pure AH Plus Jet was given and next to it its dilution, discarding the pipette tip at each use throughout the experiment. Similarly, the culture medium for the BCH and BCS groups was replaced with the osteogenic medium in that order. The experiment was performed in triplicate. The 96-well plates were again placed in an incubator at 37°C and 5% CO _2_ , where they remained for 24, 48, and 72 hours to perform the cytotoxicity assay.

**Fig. 2:**
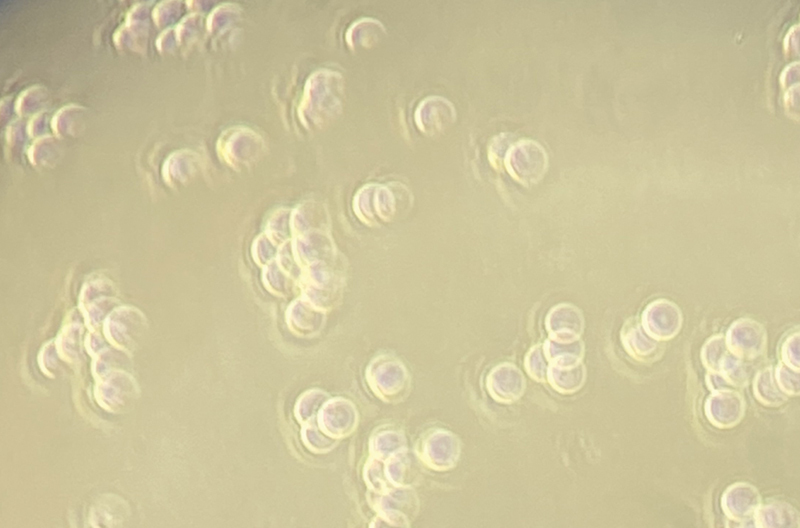
Representative image of viable cells for the cytotoxicity assay.

The viability of the cells in the presence of various eluates of endodontic sealers was evaluated using the 5 mg/mL MTT assay (Cell Proliferation Kit, Roche, Vienna, Austria). SAOS cells that had already been plated out and incubated for 24, 48, and 72 hours were each removed from the incubator to perform the assay. After each incubation period, the medium in the 96-well plate containing the conditioned sealer extract was removed, 90 µL of the medium and 10 µL of the MTT preparation, totaling 100 µL, were added to the wells, and the incubator was incubated at 37°C and 5% CO _2_ for 3 hours and counted in a chronometer to allow the MTT to be degraded from the viable cells. After the indicated time, the 96-well plate was removed from the incubator and placed again in the laminar flow chamber, where the MTT was removed and 100 µL of dimethyl sulfoxide (Sigma-Aldrich, Saint Louis, United States) was added to each well to dissolve the formazan and waited for 15 minutes. The plate was then read with a digital spectrophotometer (Epoch; Bio-Tek, Winooski, Vermont, United States) with absorbance at a wavelength of 570 nm ( Fig. 2 ).

### Alkaline Phosphatase Assay

For alkaline phosphatase (ALP) activity, the Fast Red assay (Abcam, Cambridge, UK) was used. With the extracts of each sealer already obtained, the cells were plated out in 24-well culture plates and maintained in a 37°C incubator at 5% CO _2_ . Then, the cells were exposed to the conditioned extracts at a dilution of 1:5. In this way, each activity was evaluated in a specific period, 7 and 14 days, using the Fast Red method. After each experimental period, the culture medium in each well was removed and the wells were washed at 37°C with Hanks' balanced solution. Then, 120 mM Tris buffer solution with a pH of 8.4 containing 1.8 mM Fast Red TR, 0.9 mM naphthol ASMX phosphate (Sigma-Aldrich, Saint Louis, United States), and 1:9 dimethylformamide was added to 1 mL/well. Immediately, the plates were kept in a humidified atmosphere containing 5% CO _2_ for 30 minutes. Then, 280 µL of 10% acetic acid was added to each well and the plates were shaken at room temperature for 30 minutes. The contents of each plate were then transferred to polypropylene tubes and heated to 85°C for 10 minutes. The tubes were then placed on crushed ice for 5 minutes and counted using a digital chronometer. After this time, the tubes were centrifuged at 20,000 g for 15 minutes. Then, 100 µL of the supernatant was transferred to 96-well plates along with 40 µL of 10% ammonium hydroxide to neutralize the acid. The absorbance was measured using a spectrophotometer (Epoch, Bio-Tek, Winooski, Vermont, United States) at a wavelength of 405 nm.

### Statistical Analysis

Data obtained from the experiments were analyzed using IBM SPSS software (version 26.0, IBM Corporation, Armonk, New York, United States), and graphs were generated using GraphPad Prisma software (version 8.0.0, GraphPad Software, San Diego, California, United States). The Shapiro–Wilk test was performed to verify that the data were normally distributed. Subsequently, data from the MTT assay for each dilution were compared between groups at each of the evaluation time points (24, 48, and 72 hours) by the one-way analysis of variance (ANOVA) test with Tukey's multiple comparisons posttest and over time for each of the experimental groups by the repeated measures ANOVA test and Sidak's multiple comparisons post-test. For comparisons between dilutions at the same evaluation time point and in the same group, Student's *t* -test for independent samples was used. Data from the ALP assays were compared at each of the time points (7 and 14 days) between experimental groups using the one-way ANOVA test and for each group at the different time points using the paired Student's *t* -test. A significance level of 5% was assumed for all analyzes.

## Results

The sealers differed at the assessed time points in each dilution ( Table 2 ). In both dilutions, AH Plus Jet achieved similar MTT values to the control group at 24 and 48 hours. In the first 48 hours, the BCH and BCS groups without dilution (D0) differed from each other, from the control group, and from the AH Plus Jet and were more cytotoxic. After 72 hours in D0, all sealers showed different results, when BCH and BCS had the higher cytotoxicity than AH Plus Jet, and the lowest MTT value was obtained for the BCS group in diluted form (D1) and was not different from AH Plus Jet.

**Table 2:** Comparison of values obtained for MTT between dilution, evaluation time, and experimental group

Time	Group	D0		D1		
		Mean (SD)	*p* a	Mean (SD)	*p* a	*p* b
24 h	C	0.66 (0.06) ^Aa^	<0.001	0.66 (0.06) ^Aba^	0.030	1.000
	AH Plus Jet	0.65 (0.02) ^Aa^		0.68 (0.06) ^Aba^		0.526
	BCH	0.39 (0.01) ^Ba^		0.50 (0.06) ^Aa^		0.039
	BCS	0.10 (0.03) ^Ca^		0.69 (0.08) ^Ba^		<0.001
48 h	C	0.92 (0.01) ^Ab^	<0.001	0.92 (0.01) ^Aab^	<0.001	1.000
	AH Plus Jet	0.87 (0.12) ^Aa^		0.78 (0.02) ^Ab^		0.298
	BCH	0.47 (0.04) ^Bb^		0.56 (0.11) ^Bb^		0.278
	BCS	0.15 (0.05) ^Cb^		0.88 (0.02) ^Aa^		<0.001
72 h	C	1.13 (0.14) ^Ab^	<0.001	1.13 (0.14) ^Ab^	0.003	1.000
	AH Plus Jet	0.77 (0.13) ^Ba^		0.78 (0.19) ^AB ab^		0.912
	BCH	0.39 (0.03) ^Ca^		0.51 (0.11) ^Bab^		0.150
	BCS	0.09 (0.02) ^Da^		0.62 (0.04) ^Ba^		<0.001

Abbreviations: ANOVA, analysis of variance; D0, no dilution; D1, 1:5 dilution; SD, standard deviation.

a One-way ANOVA test with Tukey posttest (Capital letters indicate comparison in the same period between sealers. Lower case letters indicate comparison of the same group in the 24h, 48h, and 72h periods by the repeated measures. ANOVA test and the Sidak's test for multiple comparisons.

b Student's *t* -test for independent samples.

Therefore, BCH and BCS were similar in MTT test mean values compared with the control group ( *p* = 0.003). AH Plus Jet was comparable to both the control group and the other sealers. When the groups were evaluated separately in different time intervals, AH Plus Jet in diluted form (D1) showed higher viability at 48 hours than at 24 hours. BCH showed the highest viability at 48 hours in both D0 and D1. Although BCS showed the lowest viability at 48 hours in D0, this condition was balanced in D1. In general, the control group values were most similar in all situations, namely AH Plus Jet followed by BCS (D1) ( Table 2 ). The ALP comparison results between groups showed a difference only after 14 days. The BCH group had the lowest values, which differed only from those of the control group. For each of the groups, no significant differences were found between the 7- and 14-day periods ( Table 3 ).

**Table 3:** Comparison between the values for alkaline phosphatase according to the time of assessment and experimental group

Time	Group	Average (SD)	*p*
7 d	C	0.16 (0.03) ^Aa^	0.057
	AH Plus Jet	0.17 (0.03) ^Aa^	
	BCH	0.11 (0.02) ^Aa^	
	BCS	0.13 (0.03) ^Aa^	
14 d	C	0.25 (0.07) ^Aa^	0.014
	AH Plus Jet	0.18 (0.02) ^ABa^	
	BCH	0.09 (0.01) ^Ba^	
	BCS	0.15 (0.02) ^Aba^	

Abbreviation: ANOVA, analysis of variance; SD, standard deviation.

Notes: One-way ANOVA test with Tukey posttested (capital letters denote comparison in the same period between sealers). Lowercase letters denote comparison of the same sealer type between the 7- and 14-day periods by the paired *t* -test.

## Discussion

Calcium silicate bioceramic are considered promising sealers due to their biocompatibility, bioactivity, and induction of hydroxyapatite formation. 18 In this study the biocompatibility and osteogenic potential in relation to osteoblastic cells of EndoSequence HiFlow and BCS were investigated in comparison with AH Plus Jet, which is considered the gold standard due to its chemical–physical properties. 7 9 19 20

Periapical extrusions associated with obturation materials are relatively common and can cause an inflammatory response and affect the success rate of clinical treatments. 19 21 Compared with other materials, CSBCs have shown a prominent role in differential mineralization of human osteoblast cells with increased calcium deposition, indicating their bioactive potency. 20 22

An important consideration in cytotoxicity testing is the choice of cell type and tissue type to be affected by the test agent. 23 Differences in cytocompatibility responses may occur between cells of different origins when exposed to endodontic sealers. 24 BioRoot RCS had a positive effect on cell metabolism of both periodontal ligaments' cells and osteoblasts. 25 In osteoblast-like cells, iRoot was found to be nontoxic with moderate cytotoxicity in mouse osteoblasts. In the present study, EndoSequence BC was strongly cytotoxic in MC3T3-E1 cells after 24 hours and became moderately cytotoxic after 6 weeks, but for most other results, EndoSequence BC and iRoot showed little or no cytotoxicity in various eluates. 26 The osteoblastic cell line, characterized by high ALP, synthesis of bone-specific proteins such as bone sialoprotein and osteocalcin, and the ability to form a mineralized layer, 25 has been the focus of studies such as MC3-T3 E1, 20 IDG-SW3, 2 and SAOS-2. 27 The present work investigated SAOS-2, which resembles human osteoblasts and has been used to test new dental materials to evaluate biocompatibility and bioactivity. 27 28 29 30

Colorimetric assays are proposed to evaluate the relationship between the viability of the tested material and the cells. In this study, an *in vitro* direct contact assay was performed according to ISO 10993-5, the colorimetric assay with MTT, which is rapid and quantitative and measures only living cells. These cells can metabolize the generated formazan as a by-product, because they have an active metabolism, which allows the evaluation of cytocompatibility. 31

In this study, BCH and BCS in undiluted form (D0) were found to have lower cell viability compared with the control group and AH Plus Jet for 24 and 48 hours, respectively, which is consistent with the studies by Loushine et al 32 and Kebudi Benezra et al. 31 After 72 hours, the BCH and BCS continued to exhibit lower cell viability, with the BCS exhibiting the highest cytotoxicity. This is in contradiction with López-García et al, 23 who reported that iron and tungsten oxide, which are present in the chemical composition of this sealer, could affect cell viability. 7

In a dilution of 1:5 (D1), BCH and BCS were similar, and AH Plus Jet was closest to the control. Significant differences between D0 and D1 dilutions were observed at 24 hours for BCH and at 48 and 72 hours for BCS. All sealers were associated with higher cell viability at dilution. Serial dilutions in *in vitro* studies following the ISO 10993-5 standardization for cytotoxicity testing facilitate the evaluation of the effects of extracts on cells from which the compounds exit and are a way to attempt to express the clinical situation of passage of tissue fluids near the foramen, where this material would be exposed. 24

The difference in cytotoxicity between the dilutions in this study may be related to their concentrations. It was found that the lower the dilution of the sealers, the lower the viability of the cells. In addition, the literature shows a time-dependent relationship of the sealers, i.e., with the passage of hours, some studies have shown a decrease in the cytotoxicity of the tested material. 21 24 32 Zhou et al 33 showed that MTA Fillapex and AH Plus Jet decreased cytotoxicity compared with control when included in diluted extracts. Fresh BCS, exhibited moderate toxicity at high concentrations, and over the weeks, both fresh and fully set extracts of AH Plus Jet and BCS no longer exhibited cytotoxicity. In agreement with this work, other authors 2 17 27 also showed that the sealers evaluated became less cytotoxic at higher dilutions. Most of the cytotoxicity tests of the sealers were performed after the material had cured. 7 16 20 In this work, as reported in previous work, 7 16 17 24 only the AH Plus Jet was cured, even after the material had been cured for 48 hours, which may have affected the results.

In the results of this study, it was found that AH Plus Jet was associated with higher cell viability compared with BCS and BCH and was closer to control, confirming the study by previous study. 32 This occurred mainly in undiluted form and differs from the results of other studies, 2 7 20 26 in which CSBC showed little or no cytotoxicity compared with AH Plus Jet. Since this sealer was the only one that could set, the result found may be related to this difference in setting. Most conventional sealers have shown insufficient biological activity in cell cultures, 7 and the cytotoxic response of AH Plus Jet is well documented, with lower toxicity values after curing and high cytotoxicity in the freshly mixed state. 21 33 34

Considering the results of the present study, it was concluded that the null hypothesis was rejected, because AH Plus Jet sealer was more viable for OSAS cells at the times tested, and the biocompatibility of calcium silicate-based sealers was acceptable.

Premixed CSBCs are hydrophilic, meaning, they rely on external moisture (canal moisture/body fluid) for their setting reaction to occur. 19 35 36 Therefore, in this work, a moist gauze was used for the CSBC to allow the setting reaction to occur, as suggested by Chen et al. 17 After 48 hours, BCS was cured in most slices compared to CSBC, whereas BCH was not cured. Curing of CSBC has been reported with varying results, 26 28 32 37 emphasizing a longer curing time, including cases where no curing occurred *in vitro* . Silva et al 36 attributed the results of lower cell viability of CSBC to this incomplete curing. In this study, our methodology did not allow the use of BCH, as recommended by the manufacturer, as a thermoplastic obturation, which may have influenced the results. 21 33 34

The difficulties to simulate the curing of CSBC in the laboratory could be an important reason for the different results in the literature. Therefore, standardization of a methodological model that attempts to achieve this curing by extracting moisture as it occurs clinically may be useful for future testing.

Bioactivity may be related to cellular effects induced by biologically active substances and CSBCs, which can release calcium ions that have the potential to influence the regenerative/interactive response 38 by forming interfacial interactions with the surrounding tissue. 22 The results of the comparison of ALP between groups showed a difference only at the second time point of the assessment, which was not significant. This result is consistent with previous results 27 28 in which the activity of ALP was observed at the second time point of the study. This result in relation to the CSBC and the control could be due to the release of calcium ions from this material.

The formation of calcium hydroxide from CSBC occurs during the initial phase of the setting process due to the release of Ca+ ions that stimulate biomineralization. 34 This process may become more noticeable after 7 to 14 days and show continuous release over a long period after setting. 35 37 Knowing that calcium ions play an important role in apatite formation and that the activity of ALP is an early marker of osteoblast maturation, Lee et al 20 found that periods of up to 7 days may be opportune when using these sealers in terms of osteogenic effect.

A possible limitation of the present results is that it is an *in vitro* study, may not reproduce the same clinical conditions, and may introduce bias. Nonetheless, they are necessary to be carried out and may be clinically applicable in the future to establish clinical protocols so they should always be encouraged and improved.

The results of this study suggest that the biocompatibility of CSBS, such as BCS, EndoSequence BCH, and AH Plus Jet, is acceptable, and the effects on cell viability depend on dilution concentration and exposure time, and they have osteogenic effect and bioactivity.

## References

[1] Grossman L I An improved root canal cement J Am Dent Assoc 1958 56 03 381 385 13513298 10.14219/jada.archive.1958.0055

[2] Giacomino C M Wealleans J A Kuhn N Diogenes A Comparative biocompatibility and osteogenic potential of two bioceramic sealers J Endod 2019 45 01 51 56 30558798 10.1016/j.joen.2018.08.007

[3] Cintra L TA Benetti F de Azevedo Queiroz ÍO Evaluation of the cytotoxicity and biocompatibility of new resin epoxy-based endodontic sealer containing calcium hydroxide J Endod 2017 43 12 2088 2092 29032822 10.1016/j.joen.2017.07.016

[4] Antunes T BM Janini A CP Pelepenko L E Heating stability, physical and chemical analysis of calcium silicate-based endodontic sealers Int Endod J 2021 54 07 1175 1188 33577106 10.1111/iej.13496

[5] Chybowski E A Glickman G N Patel Y Fleury A Solomon E He J Clinical outcome of non-surgical root canal treatment using a single-cone technique with EndoSequence bioceramic sealer: a retrospective analysis J Endod 2018 44 06 941 945 29606401 10.1016/j.joen.2018.02.019

[6] Yan Y Li Y Chi Y Ji M Shen Y Zou L A comparative study of biological properties of three root canal sealers Clin Oral Investig 2023 28 01 11 10.1007/s00784-023-05402-7 38129367

[7] Rodríguez-Lozano F J García-Bernal D Oñate-Sánchez R E Ortolani-Seltenerich P S Forner L Moraleda J M Evaluation of cytocompatibility of calcium silicate-based endodontic sealers and their effects on the biological responses of mesenchymal dental stem cells Int Endod J 2017 50 01 67 76 26660310 10.1111/iej.12596

[8] Penha da Silva P J Marceliano-Alves M F Provenzano J C Dellazari R LA Gonçalves L S Alves F RF Quality of root canal filling using a bioceramic sealer in oval canals: a three-dimensional analysis Eur J Dent 2021 15 03 475 480 33535249 10.1055/s-0040-1722095 PMC8382469

[9] Lim E S Park Y B Kwon Y S Shon W J Lee K W Min K S Physical properties and biocompatibility of an injectable calcium-silicate-based root canal sealer: in vitro and in vivo study BMC Oral Health 2015 15 01 129 26490372 10.1186/s12903-015-0112-9 PMC4618726

[10] Rodríguez-Lozano F J López-García S García-Bernal D Chemical composition and bioactivity potential of the new EndoSequence BC Sealer formulation HiFlow Int Endod J 2020 53 09 1216 1228 32412113 10.1111/iej.13327

[11] Hage W Sarkis D K Kallasy M Antimicrobial activity of five calcium silicate based root canal sealers against a multispecies engineered biofilm: an *in vitro* study J Contemp Dent Pract 2023 24 09 707 714 38152946 10.5005/jp-journals-10024-3556

[12] Quaresma S AL Alves Dos Santos G N Silva-Sousa A C Physicochemical properties of calcium silicate cement based endodontic sealers J Mech Behav Biomed Mater 2024 151 106400 38262184 10.1016/j.jmbbm.2024.106400

[13] Sanz J L Guerrero-Gironés J Pecci-Lloret M P Pecci-Lloret M R Melo M Biological interactions between calcium silicate-based endodontic biomaterials and periodontal ligament stem cells: a systematic review of in vitro studies Int Endod J 2021 54 11 2025 2043 34338339 10.1111/iej.13600

[14] Kandemir Demirci G Çöven F O Güneri P The solubility, pH value, chemical structure, radiopacity, and cytotoxicity of four different root canal sealers: an in vitro study Clin Oral Investig 2023 27 09 5413 5425 10.1007/s00784-023-05160-6 37486382

[15] Myong-hyun B Lozano A Forner L Llena C López-García .pdf 2019 10.1007/s00784-019-03036-2 31399829

[16] Vouzara T Dimosiari G Koulaouzidou E A Economides N Cytotoxicity of a new calcium silicate endodontic sealer J Endod 2018 44 05 849 852 29550005 10.1016/j.joen.2018.01.015

[17] Chen B Haapasalo M Mobuchon C Li X Ma J Shen Y Cytotoxicity and the effect of temperature on physical properties and chemical composition of a new calcium silicate-based root canal sealer J Endod 2020 46 04 531 538 32081458 10.1016/j.joen.2019.12.009

[18] Zordan-Bronzel C L Tanomaru-Filho M Rodrigues E M Chávez-Andrade G M Faria G Guerreiro-Tanomaru J M Cytocompatibility, bioactive potential and antimicrobial activity of an experimental calcium silicate-based endodontic sealer Int Endod J 2019 52 07 979 986 30702145 10.1111/iej.13086

[19] Kim J H Cho S Y Choi Y Kim D H Shin S J Jung I Y Clinical efficacy of sealer-based obturation using calcium silicate sealers: a randomized clinical trial J Endod 2022 48 02 144 151 34856212 10.1016/j.joen.2021.11.011

[20] Lee B N Hong J U Kim S M Anti-inflammatory and osteogenic effects of calcium silicate-based root canal sealers J Endod 2019 45 01 73 78 30558800 10.1016/j.joen.2018.09.006

[21] Candeiro G TM Moura-Netto C D'Almeida-Couto R S Cytotoxicity, genotoxicity and antibacterial effectiveness of a bioceramic endodontic sealer Int Endod J 2016 49 09 858 864 26281002 10.1111/iej.12523

[22] Donnermeyer D Bürklein S Dammaschke T Schäfer E Endodontic sealers based on calcium silicates: a systematic review Odontology 2019 107 04 421 436 30554288 10.1007/s10266-018-0400-3

[23] López-García S Pecci-Lloret M R Guerrero-Gironés J Comparative cytocompatibility and mineralization potential of Bio-C sealer and totalfill BC sealer Materials (Basel) 2019 12 19 1 12 10.3390/ma12193087 PMC6804055 31546696

[24] Collado-González M García-Bernal D Oñate-Sánchez R E Biocompatibility of three new calcium silicate-based endodontic sealers on human periodontal ligament stem cells Int Endod J 2017 50 09 875 884 27666949 10.1111/iej.12703

[25] Majors A K Boehm C A Nitto H Midura R J Muschler G F Characterization of human bone marrow stromal cells with respect to osteoblastic differentiation J Orthop Res 1997 15 04 546 557 9379264 10.1002/jor.1100150410

[26] Silva Almeida L H Moraes R R Morgental R D Pappen F G Are premixed calcium silicate-based endodontic sealers comparable to conventional materials? A systematic review of in vitro studies J Endod 2017 43 04 527 535 28216270 10.1016/j.joen.2016.11.019

[27] Tanomaru-Filho M Andrade A S Rodrigues E M Biocompatibility and mineralized nodule formation of Neo MTA Plus and an experimental tricalcium silicate cement containing tantalum oxide Int Endod J 2017 50 02 e31 e39 28390072 10.1111/iej.12780

[28] Zordan-Bronzel C L Esteves Torres F F Tanomaru-Filho M Chávez-Andrade G M Bosso-Martelo R Guerreiro-Tanomaru J M Evaluation of physicochemical properties of a new calcium silicate-based sealer, Bio-C Sealer J Endod 2019 45 10 1248 1252 31447172 10.1016/j.joen.2019.07.006

[29] Yang X Tian J Li M Biocompatibility of a new calcium silicate-based root canal sealer mediated via the modulation of macrophage polarization in a rat model Materials (Basel) 2022 15 05 1962 35269193 10.3390/ma15051962 PMC8911908

[30] Saber S Raafat S Elashiry M El-Banna A Schäfer E Effect of different sealers on the cytocompatibility and osteogenic potential of human periodontal ligament stem cells: an in vitro study J Clin Med 2023 12 06 2344 36983344 10.3390/jcm12062344 PMC10056919

[31] Kebudi Benezra M Schembri Wismayer P Camilleri J Interfacial characteristics and cytocompatibility of hydraulic sealer cements J Endod 2018 44 06 1007 1017 29398087 10.1016/j.joen.2017.11.011

[32] Loushine B A Bryan T E Looney S W Setting properties and cytotoxicity evaluation of a premixed bioceramic root canal sealer J Endod 2011 37 05 673 677 21496669 10.1016/j.joen.2011.01.003

[33] Zhou H M Du T F Shen Y Wang Z J Zheng Y F Haapasalo M In vitro cytotoxicity of calcium silicate-containing endodontic sealers J Endod 2015 41 01 56 61 25442721 10.1016/j.joen.2014.09.012

[34] Sfeir G Zogheib C Patel S Giraud T Nagendrababu V Bukiet F Calcium silicate based root canal sealers: a narrative review and clinical perspectives Materials (Basel) 2021 14 14 3965 34300886 10.3390/ma14143965 PMC8306764

[35] Zamparini F Siboni F Prati C Taddei P Gandolfi M G Properties of calcium silicate-monobasic calcium phosphate materials for endodontics containing tantalum pentoxide and zirconium oxide Clin Oral Investig 2019 23 01 445 457 10.1007/s00784-018-2453-7 29737429

[36] Silva E JNL Ehrhardt I C Sampaio G C Determining the setting of root canal sealers using an in vivo animal experimental model Clin Oral Investig 2021 25 04 1899 1906 10.1007/s00784-020-03496-x 32789655

[37] Prati C Gandolfi M G Calcium silicate bioactive cements: biological perspectives and clinical applications Dent Mater 2015 31 04 351 370 25662204 10.1016/j.dental.2015.01.004

[38] Duarte M AH Marciano M A Vivan R R Tanomaru Filho M Tanomaru J MG Camilleri J Tricalcium silicate-based cements: properties and modifications Braz Oral Res 2018 32 01 e70 30365611 10.1590/1807-3107bor-2018.vol32.0070

